# Diagnostic odyssey and healthcare access challenges in Rett Syndrome: perspectives from Brazilian families

**DOI:** 10.1590/1984-0462/2026/44/2025329

**Published:** 2026-09-25

**Authors:** Gabriella Almeida Santos de Santana, Lindinês de Jesus Sousa, Maria José Souza Silva, Narciso DiLorenzo Machado Dias, Gabriela Garcia de Carvalho Laguna, Larissa Alves Fernandes, Cintia Rodrigues Marques, Patrícia da Silva Oliveira

**Affiliations:** a Universidade Federal da Bahia, Vitória da Conquista, BA, Brasil.

**Keywords:** Rett Syndrome, Delayed diagnosis, Caregiver burden, Rare diseases, Qualitative research, Health services accessibility, Síndrome de Rett, Diagnóstico tardio, Sobrecarga do cuidador, Doenças raras, Acesso aos serviços de saúde

## Abstract

**Objective:**

To analyze the challenges in the diagnosis and care of Rett Syndrome based on the experiences of caregivers in Brazil.

**Methods:**

This study employed a convergent mixed-methods approach. Data were collected from 14 primary caregivers of children with genetically confirmed Rett Syndrome. Data collection included thematic analysis of semi-structured interviews and descriptive statistics from sociodemographic questionnaires.

**Results:**

The mean diagnostic delay was 3.6 years (standard deviation — SD=1.4 years; range, 2–6 years), with a median of 3.5 years. The distribution was assessed and deemed suitable for reporting the mean. Most cases (92.8%) were initially attributed to autism spectrum disorder. The main barriers were limited knowledge among professionals (78.6%), delays in genetic testing (92.8%), and difficulties accessing therapies. Caregivers (12 mothers, 2 fathers; mean age, 38.0 years [SD, 4.1]; 57.1% with a college degree) reported a significant physical and emotional burden, with 92.8% providing full-time care without institutional support. The qualitative analysis revealed four themes: the diagnostic journey, the battle for therapy, the impact on family life, and the burden of care.

**Conclusions:**

Diagnosis and care are hindered by knowledge gaps, systemic inefficiencies, and limited support. The findings highlight the need to improve education on rare diseases, streamline access to genetic testing and therapies, and implement support programs for caregivers.

## RESUMO

**Objetivo:**

Analisar os desafios no diagnóstico e cuidado da Síndrome de Rett com base nas experiências de cuidadores no Brasil.

**Métodos:**

Este estudo empregou um delineamento de métodos mistos convergentes, com 14 cuidadores primários de crianças com síndrome de Rett geneticamente confirmada. A coleta de dados incluiu a análise temática de entrevistas semiestruturadas e a análise estatística descritiva de questionários sociodemográficos.

**Resultados:**

O atraso diagnóstico médio foi de 3,6 anos (desvio padrão — DP=1,4 anos; variação, 2–6 anos), com mediana de 3,5 anos; a distribuição foi considerada adequada para o uso da média. A maioria dos casos (92,8%) foi inicialmente atribuída ao transtorno do espectro autista. As principais barreiras foram: conhecimento limitado dos profissionais (78,6%), atrasos no teste genético (92,8%) e dificuldades de acesso a terapias. Os cuidadores (12 mães, 2 pais; idade média, 38,0 anos [DP, 4,1]; 57,1% com ensino superior completo) relataram uma sobrecarga física e emocional significativa, com 92,8% prestando cuidados em tempo integral sem apoio. A análise qualitativa revelou quatro temas: a jornada diagnóstica, a batalha por terapia, o impacto na vida familiar e a sobrecarga do cuidado.

**Conclusões:**

O diagnóstico e cuidado são dificultados por lacunas no conhecimento, ineficiências sistêmicas e suporte limitado. Os resultados destacam a necessidade de melhorar a educação sobre doenças raras, agilizar o acesso a testes genéticos e terapias e implementar programas de apoio aos cuidadores.

## INTRODUCTION

In 1966, Andreas Rett first described a neurological disorder characterized by psychomotor developmental delay, ataxia, hand stereotypies, and seizures, later named Rett syndrome (RTT).^1^ The discovery of mutations in the MECP2 gene (methyl-CpG binding protein 2) in 1999 revolutionized the diagnosis and spurred research into RTT.^2,3^ Today, RTT is recognized as a rare, X-linked dominant disorder, predominantly affecting females (XX), with an estimated prevalence of 1:10,000 girls and 1:1,000,000 boys.^4^

Approximately 90% of cases involve mutations in MECP2, which encodes a protein critical for gene expression regulation and proper neurological development.^3,4^ These mutations are typically sporadic, with no defined hereditary pattern or parental inheritance correlation. Additionally, random X-chromosome inactivation in females leads to variable phenotypic expression, complicating genotype-phenotype correlations.^5^

Clinically, RTT follows a predictable four-stage course, as summarized in Table 1.^6,7^ The first stage, early stagnation, is characterized by subtle slowing of neuropsychomotor development and reduced social interest. The second stage, rapid regression, is the most diagnostically distinctive, involving loss of purposeful hand use and expressive language, emergence of hand stereotypies, and behaviors that may mimic ASD. The third stage, the pseudostationary phase, is marked by relative clinical stabilization, with some improvement in social engagement despite persistent motor and cognitive deficits. Finally, the fourth stage corresponds to late motor deterioration, with progressive loss of mobility, muscle rigidity, and scoliosis, alongside relative preservation of nonverbal social interaction.^2,4^

**Table 1:** Clinical Stages of Rett Syndrome.

Stage	Age range	Key characteristics
Stage 1: Early Onset (Stagnation)	6–18 months	Apparently normal early development Subtle signs: developmental delays (postural control, motor skills, language) Reduced eye contact, hand-wringing may emerge Microcephaly may begin
Stage 2: Rapid Regression	1–3 years	Loss of acquired skills: purposeful hand use, spoken language Stereotypical hand movements (wringing, tapping) Autistic-like behaviors, unsteady gait Breathing irregularities (apnea/hyperventilation) Seizures may onset
Stage 3: Plateau	3–10 years	Stabilization of regression Possible improvement in communication/social engagement Persistent seizures Motor difficulties (apraxia/dyspraxia) Progressive microcephaly
Stage 4: Late Motor Deterioration	≥10 years	Severe physical decline: muscle weakness, spasticity, or rigidity Wheelchair dependency common Continued seizures Scoliosis and other skeletal deformities Long-term survival possible with supportive care

Source: Adapted from Neul et al.^6^

This regressive pattern, especially the loss of acquired motor and communication skills, represents a central feature that distinguishes RTT from ASD, which is characterized by persistent developmental differences rather than regression. Symptoms typically emerge between six and 18 months of age, often after seemingly normal early development. Subsequently, progressive neurological decline results in loss of motor and communication skills, seizures, hand stereotypies, respiratory issues, and motor deficits. Without proper management, these symptoms can lead to severe complications, including intellectual disability, scoliosis, feeding disorders, and sphincter dysfunction.^6,7^

Early diagnosis is crucial for initiating effective therapeutic interventions, such as physical therapy, speech therapy, occupational therapy, and assistive technologies. The pharmacological landscape is evolving globally, with the approval of medications like trofinetide (Daybue^®^) in 2023 in other countries, opening new perspectives for symptom relief and improved quality of life.^8,9^ Promisingly, novel drug repurposing approaches using artificial intelligence (AI) and network analysis have also identified candidates like vorinostat, which has shown efficacy in preclinical models for ameliorating multi-system abnormalities in RTT, even when administered after symptom onset.^10^ Primary healthcare plays a pivotal role in early recognition, diagnostic referral, care coordination, and facilitating access to rehabilitation and support services, which are essential for preventing skeletal deformities and enhancing mobility and social development.^11,12^

Given the complexity of RTT, a multidisciplinary approach is required to address the needs of patients and their families, who face ongoing physical, socioeconomic, and emotional challenges. However, the specific nature of the barriers faced by families in navigating the healthcare system for diagnosis and ongoing care remains poorly characterized, particularly in the Brazilian context. This study, therefore, aimed to answer the following question: What are the primary challenges faced by Brazilian families during the diagnostic journey and in accessing healthcare for Rett syndrome? By analyzing caregivers’ experiences, we seek to provide clinical insights while amplifying the voices of those directly affected.

## METHOD

This descriptive, exploratory study employed a convergent mixed-methods design, in which quantitative and qualitative data were collected in parallel, analyzed separately, and then integrated during the interpretation phase through methodological triangulation to provide a comprehensive understanding of caregiver experiences.

Participants were recruited from a national virtual support group for Rett Syndrome on WhatsApp and Instagram. Snowball sampling was subsequently employed within this network, whereby initial participants were invited to refer other eligible caregivers known to them, until data saturation was reached.^13^ This recruitment strategy was chosen due to the rarity of the syndrome; however, it may introduce selection bias, as caregivers engaged in support networks might have greater access to information, resources, or advocacy experiences compared to those not connected to such groups. This potential bias should be considered when generalizing the findings. Inclusion criteria required participants to be primary caregivers of children or adolescents with a clinical and genetically confirmed diagnosis of Rett Syndrome (via MECP2 gene analysis). The final sample size was 14 primary caregivers, determined by reaching data saturation in the qualitative component.

Data collection was conducted from February 2024 to October 2024 using two primary instruments: semi-structured interviews and questionnaires. The interviews were conducted remotely via Google Meet, with an average duration of 60 minutes, and were guided by a script covering multidimensional aspects, including diagnostic trajectory, healthcare access, support networks, daily challenges, and future perspectives. Additionally, a sociodemographic form and a Rett Syndrome-specific questionnaire were administered to characterize the sample. This instrument was developed for this study by the research team (comprising a pediatric neurologist and two public health researchers) based on clinical literature and expert input. It was pilot tested with two caregivers to ensure clarity and relevance, with minor wording adjustments made prior to data collection. All participants provided written informed consent electronically via Google Forms after being thoroughly briefed on the study’s objectives and procedures. In accordance with the informed consent form, the interviews were audio-recorded and transcribed verbatim using TurboScribe.ai software. All transcripts were subsequently reviewed and manually verified by the lead researcher to ensure accuracy prior to analysis. The interview responses were transcribed in Portuguese and, for the purpose of presentation in this article, were translated into English by the authors.

The data analysis followed a dual approach corresponding to the two types of data collected. For the qualitative data derived from the interviews, a Thematic Analysis was conducted following the methodological framework proposed by Braun and Clarke,^14^ comprising six sequential steps: familiarization with the data through repeated reading of the transcripts; generation of initial codes identifying relevant data segments; search for themes by grouping codes into potential thematic clusters; review of themes against the codes and the entire dataset; defining and naming themes by refining their essence and scope; and producing the report by selecting illustrative excerpts to support the final analysis. The analysis was performed inductively, focusing on the semantic level of the data.

For the quantitative data obtained from the questionnaires, the information was processed using IBM Statistical Package for the Social Sciences (SPSS) Statistics, version 23.0. In alignment with the descriptive aims of the study and its sample size, the analysis was confined to descriptive statistics. Quantitative variables were summarized using measures of central tendency, such as the mean and median, and measures of dispersion, including standard deviation and range. Categorical variables were analyzed and presented using absolute numbers and relative frequencies (percentages). It is noted that inferential statistical tests and power calculations were not undertaken, as they were not pertinent to the descriptive objectives of this research. Given the exploratory nature of the quantitative component and the small sample size, these descriptive statistics are presented solely to characterize the sample and should not be interpreted as population estimates.

The study protocol received prior approval from the Research Ethics Committee of the Universidade Federal da Bahia (Certificate of Presentation for Ethical Appreciation — CAAE: 83219924.8.0000.5556) and was conducted in accordance with the Brazilian CNS Resolution 466/2012. Written informed consent was obtained from all participants, with additional consent obtained for the publication of anonymized data. Participant confidentiality was maintained through data anonymization and secure storage. This study was conducted in accordance with the Strengthening the Reporting of Observational Studies in Epidemiology (STROBE) statement for cross-sectional studies.^15^

## RESULTS

The study included 14 primary caregivers (85.7% mothers; mean age, 38.0±4.1 years), caring for girls with genetically confirmed Rett Syndrome in Brazil. Demographic data indicated a mixed-race population (64.3%) from low-income households, with 50% of families earning at or below two minimum wages (Table 2). Most children had private insurance (85.7%), yet over half of caregivers (57.1%) reported that treatments are frequently denied, necessitating legal action to secure coverage.

**Table 2:** Socioeconomic profile of families (n=14).

Category	Responses (%)
Marital status	Married/stable union: 50 Single: 28.6 Divorced/separated: 21.4
Caregiver education levels	College degree: 57.1 High school/vocational: 35.7 Elementary/some college: 7.2
Self-reported ethnicity	Mixed race: 64.3 White: 21.4 Black: 14.3
Monthly family income*	≤$600: 50 $601–$1,200: 28.6 > $1,200: 21.4
Government assistance	Yes: 50 No: 50
Health insurance	Yes: 85.7 No: 14.3
Religion	Catholic: 50 Protestant: 21.5 Spiritist: 14.3 None: 7.1 Not informed: 7.1
Caregivers per child	1 caregiver: 28.6 2 caregivers: 42.8 ≥3 caregivers: 28.6
Daily care commitment	Full-time: 92.8 Part-time (school hours): 7.2
Caregiver emotional support	None: 85.7 Received (psychological/family): 14.3
Region of residence	Northeast: 57.1 Southeast: 21.4 South: 21.4

*Income values converted to USD equivalents (~R$5 = $1) for international readability.

Note: Data are presented as n (%). All variables had complete data (n=14) unless otherwise specified.

Source: Author’s own production.

Clinical evaluation showed classic symptoms depicted in Table 1, including universal motor skill loss (100%) and seizures (100%), alongside highly prevalent hand stereotypies (92.8%) and communication regression (92.8%). The path to diagnosis was notably prolonged, with an average delay of 3.6 years (standard deviation — SD=1.4 years; range, 2–6 years) from initial symptom onset to genetic confirmation. A striking 92.8% of cases were initially misdiagnosed, most commonly with autism spectrum disorder.

Regarding therapeutic interventions, while access to basic therapies like physical therapy (100%) and speech therapy (92.9%) was high, access to specialized care, such as augmentative communication therapy, remained limited (14.3%) (Figure 1). Neurological follow-up was common (92.9%), but access to occupational therapy was lower (64.3%). None of the patients had access to syndrome-specific medications like Trofinetide. Caregiver burden was substantial, with 92.8% providing full-time care without formal support. Despite this significant caregiving burden, only 14.3% of caregivers reported receiving psychological support. Common daily challenges included feeding difficulties (57.1%) and sleep deprivation (35.7%).

**Figure 1:**
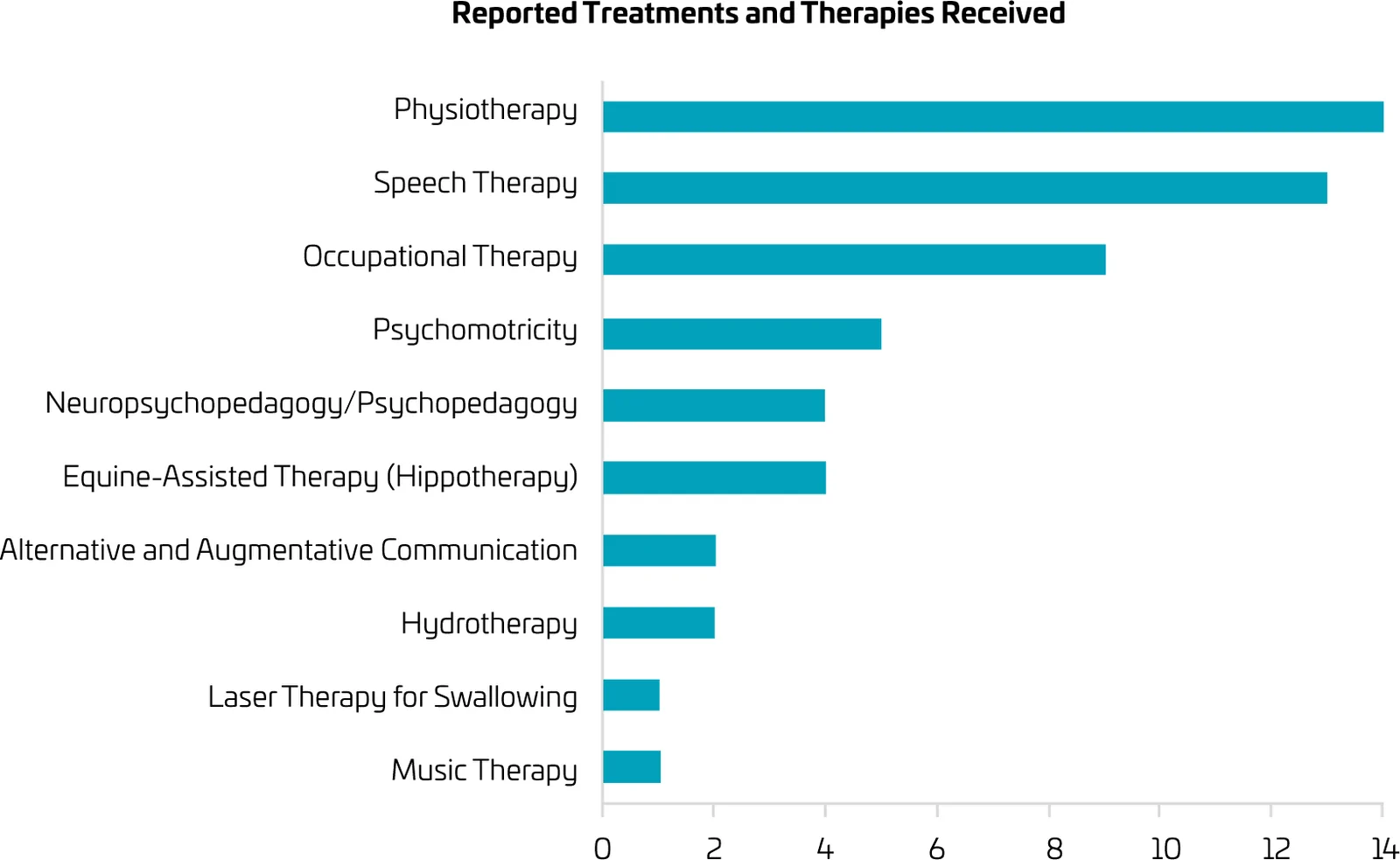
Reported Treatments and Therapies Received (n=14).

Source: Author’s own production.

Thematic analysis of caregiver interviews provided profound context to these quantitative findings, revealing four central themes that describe the complex experience of caregivers of children with Rett Syndrome in Brazil:

1. The Diagnostic Odyssey and the Struggle for Recognition: This theme encompasses the long and uncertain journey to obtain a correct diagnosis, often marked by initial misdiagnoses (such as autism spectrum disorder) and a lack of professional knowledge about Rett Syndrome. Caregivers reported feeling invalidated and having to actively educate professionals. The delay in genetic confirmation was a major source of distress, leading to a loss of critical time for early intervention. “The confusion with autism delayed everything. We lost precious time for early intervention. We had to fight for the genetic test; no one believed it was Rett.*”
2. The Battle for Therapy and Accessibility: This theme highlights the persistent struggle to secure adequate and continuous multidisciplinary therapies (physiotherapy, speech therapy, occupational therapy, etc.). Despite having health insurance, caregivers frequently faced bureaucratic barriers, denial of coverage, and the need for legal action to guarantee their children’s rights. The lack of specialized professionals and the high cost of private care were constant challenges. “It’s an exhausting battle against the health plan. Every six months, they try to cut the therapies. We have a court order, but the fight never ends.”
3. The Emotional Impact and the Transformation of Family Life: The diagnosis and the demands of care caused a profound emotional impact, including feelings of guilt, fear, and chronic stress. This theme also covers the radical transformation of family life, with caregivers (mostly mothers) abandoning their professional careers and social lives to dedicate themselves exclusively to care. The lack of psychological support for the caregiver was a recurring complaint. “My life changed completely. I had to stop working... Social life practically ended. Sometimes I feel like I’m drowning, but I have to be strong for her.”
4. Daily Care Challenges and Overload: This theme addresses the practical difficulties of daily care, including managing the complex clinical symptoms of Rett Syndrome, such as seizures, feeding difficulties, and sleep disturbances. The constant need for full-time care, often without formal institutional or family support, leads to physical and emotional overload for the primary caregiver. “The nights are the hardest. She has seizures and wakes up constantly. I haven’t had a full night’s sleep in years. The exhaustion is constant.”

The integrated results demonstrate how diagnostic failures and systemic barriers collectively exacerbate the challenges of managing a complex neurodevelopmental disorder.

## DISCUSSION

This study illuminates the multifaceted challenges faced by Brazilian families navigating the complex landscape of Rett syndrome, revealing critical gaps in diagnosis, access to care, and support systems. While caregiver burden is well-documented in other neurodevelopmental disorders,^16,17^ this study uniquely contributes by mapping the specific diagnostic odyssey and systemic barriers in the under-researched Brazilian context, using a mixed-methods approach to provide depth to the quantitative findings. Similar challenges, including diagnostic delays, therapy access barriers, and caregiver burden, have been reported in families of children with autism spectrum disorder, Dravet syndrome, and 22q11.2 deletion syndrome.^16,17,18^ However, the specific regressive pattern of RTT introduces unique diagnostic complexities that distinguish it from these conditions.^6^

The diagnostic delay observed in our Brazilian cohort (mean 3.6 years, ~43 months) was notably longer than that reported in a recent Portuguese study, where the median age at genetic confirmation was 35 months.^19^ This disparity suggests that progress toward earlier diagnosis, attributed in the Portuguese context to increased awareness and genetic testing access, has not yet been fully realized in Brazil. This underscores the fact that delays are not isolated but reflect a broader, systemic challenge in resource-limited settings. Caregiver narratives vividly illustrate this struggle, with one parent recalling, ‘The confusion with autism delayed everything. We lost precious time for early intervention.’ This diagnostic odyssey is further complicated by the complex genotype-phenotype relationships in RTT. Recent evidence indicates that specific MECP2 variants are associated with distinct behavioral phenotypes, including heightened anxiety and mood disturbances,^20^ manifestations that may be mistakenly attributed to ASD. Our findings, illuminated by caregiver experiences, highlight an urgent need for improved physician education on RTT’s distinctive regression pattern and its nuanced behavioral manifestations, which differ fundamentally from ASD’s developmental course.^6^

The clinical presentation of universal motor regression and frequent hand stereotypies confirms classic RTT phenotypes.^5,6^ Oral manifestations such as bruxism, reported by caregivers in Figure 2, are also well-documented features of RTT.^21,22^ Our qualitative accounts provide profound context to these clinical features, with one caregiver describing, ‘Her speech diminished until it completely stopped... It’s as if she had unlearned...’ Importantly, the behavioral profile in RTT extends beyond these core features. As demonstrated by Downs et al.,^23^ the Rett Syndrome Behavior Questionnaire captures a broad spectrum of challenges, including night-time behaviors and fear/anxiety, which are particularly prominent in younger individuals. Thus, our participants’ experiences reflect only one dimension of a multifaceted behavioral phenotype that requires comprehensive assessment. A critical finding was the stark disparity between the universal access to basic therapies, as indicated by quantitative data, and the exhausting battles families described to secure adequate intervention intensity, often resorting to legal action for insurance coverage.^24^ This chasm between technical availability and practical access to care underscores systemic inefficiencies inherent in rare disease management.

**Figure 2:**
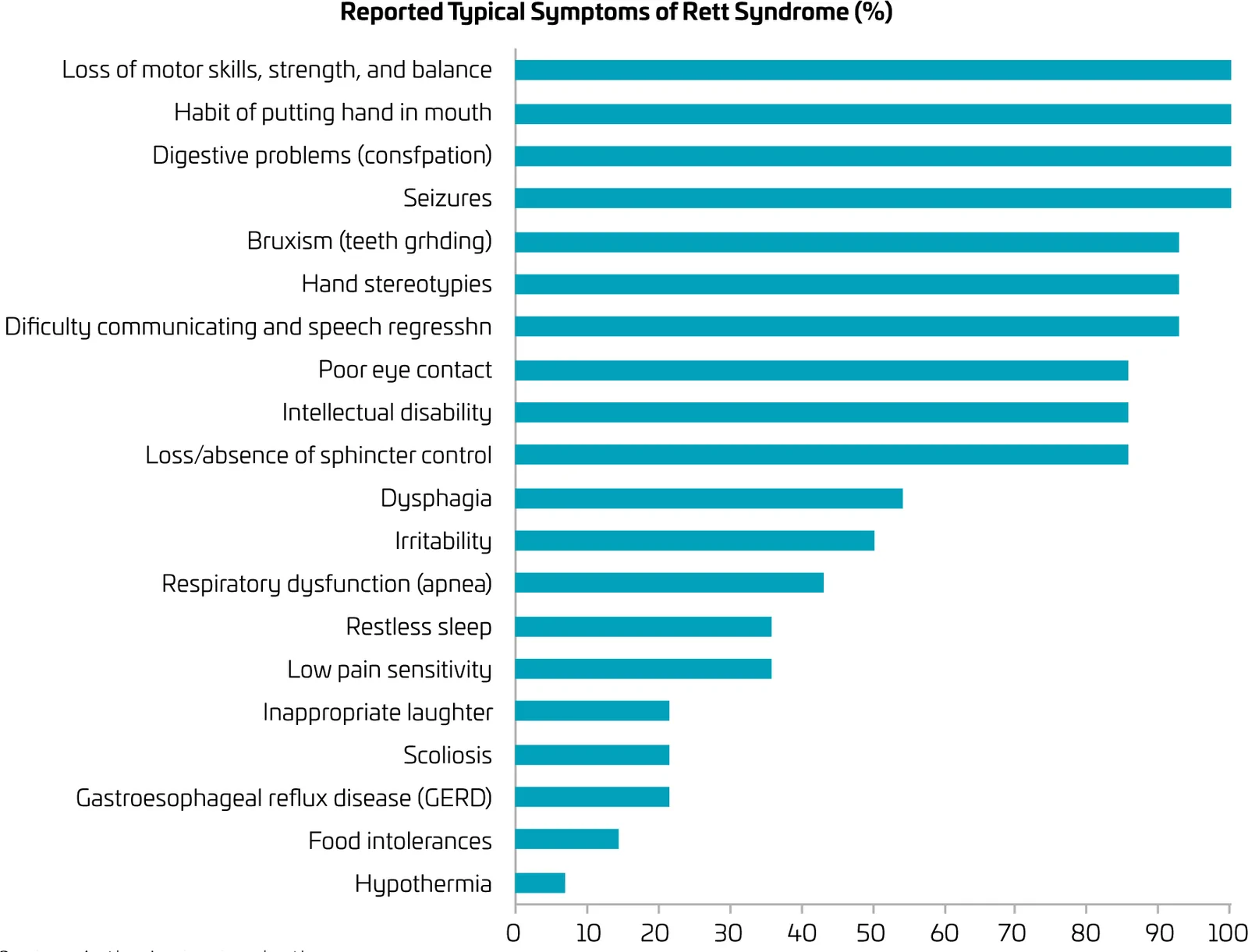
Reported Typical Symptoms of Rett Syndrome (n=14).

Source: Author’s own production.

Socioeconomic status and access to specialized services act as primary determinants of the diagnostic and therapeutic journey in Brazil. The ability to access reference centers and specialized professionals is decisive for obtaining a timely diagnosis; its absence significantly prolongs the diagnostic odyssey. Qualitative research consistently identifies practical barriers, such as a lack of local specialists, transportation difficulties, and the high cost of treatments, that disproportionately affect families dependent on the public system (SUS), where long wait times and limited-service availability are common. Conversely, possessing the means to access private healthcare or travel to specialized centers serves as a crucial buffer, reducing these systemic barriers.^16^ National studies on specific rare genetic conditions, such as 22q11.2, corroborate this, showing that more favorable socioeconomic conditions are associated with earlier diagnosis, indicating a direct link between economic status and diagnostic timing in Brazil.^16,17^

While higher caregiver education may theoretically facilitate healthcare navigation, its influence in Brazil appears secondary to more decisive structural factors — socioeconomic status, type of healthcare access (private vs. SUS), and proximity to specialized services.^16,18^

The psychosocial dimension of RTT care revealed a profound caregiver burden, quantitatively captured by the finding that 92.8% of caregivers provide full-time care without formal support. However, this statistic alone fails to convey the full impact, which emerged powerfully through qualitative accounts describing the physical and emotional toll, with one caregiver noting it was ‘like taking care of a large baby all the time.’ These accounts align with established literature on caregiver burden in rare diseases,^16,20,24,25,26,27^ yet also demonstrate remarkable resilience often forged through peer support networks. Despite these challenges, families maintained forward-looking perspectives centered on hope for scientific advances and quality-of-life improvements, an approach supported by recent work on family-centered care models.^17^

These interconnected findings demonstrate how diagnostic delays compound clinical management difficulties within systems ill-equipped to support RTT’s complexities. The results suggest three key priorities for intervention: enhanced clinician training on RTT’s early signs, with particular emphasis on features distinguishing it from ASD; streamlined access to genetic testing and multidisciplinary care; and comprehensive support programs addressing both patient needs and caregiver burdens.^10,16,27^ This is particularly relevant given the rapidly evolving therapeutic landscape for RTT, which now includes the first Food and Drug Administration (FDA)-approved drug and numerous agents in clinical and preclinical development.^8,9^

This study’s strengths include its mixed-methods design, which integrated quantitative and qualitative data for a comprehensive understanding of caregiver experiences, and the inclusion of only genetically confirmed cases, ensuring diagnostic accuracy. However, the findings should be interpreted considering several limitations. First, the small sample size (n=14) and the use of snowball sampling from a single virtual support group may limit the generalizability of the findings and introduce selection bias, as caregivers engaged in support networks may have greater access to information, resources, or advocacy experiences compared to those not connected to such groups. Second, the quantitative data are descriptive and exploratory; inferential statistics were not performed, and the results should not be extrapolated to the broader population of caregivers of individuals with Rett Syndrome in Brazil. Third, reliance on self-reported data may be subject to recall bias, particularly regarding diagnostic delays and treatment histories. Finally, the cross-sectional design captures experiences at a single time point, limiting the ability to assess changes over time or long-term outcomes.

In conclusion, this study highlights critical gaps in RTT diagnosis and care in Brazil, underscoring the need for enhanced physician education on RTT’s early signs, streamlined access to genetic testing and multidisciplinary care, and comprehensive caregiver support programs. Addressing these priorities through collaborative efforts among clinicians, policymakers, and patient advocacy groups, and leveraging emerging therapeutic approaches such as AI-driven drug repurposing, is essential to improving outcomes for individuals with Rett syndrome and their families.

## Data Availability

The database that originated the article is available with the corresponding author.

## Footnotes

* The quotes presented below were translated from the original Portuguese transcripts by the authors.
